# Melatonin Treatment Inhibits the Growth of *Xanthomonas oryzae* pv. *oryzae*

**DOI:** 10.3389/fmicb.2018.02280

**Published:** 2018-10-04

**Authors:** Xian Chen, Cheng Sun, Pedro Laborda, Yancun Zhao, Ian Palmer, Zheng Qing Fu, Jingping Qiu, Fengquan Liu

**Affiliations:** ^1^Institute of Plant Protection, Jiangsu Key Laboratory for Food Quality and Safety-State Key Laboratory Cultivation Base of Ministry of Science and Technology, Jiangsu Academy of Agricultural Sciences, Nanjing, China; ^2^School of Medicine, Yangzhou Polytechnic College, Yangzhou, China; ^3^Department of Biological Sciences, University of South Carolina, Columbia, SC, United States; ^4^Institute of Botany, Jiangsu Province and Chinese Academy of Sciences, Nanjing, China

**Keywords:** melatonin, *Xanthomonas oryzae* pv. *oryzae*, antibacterial action, growth, transcriptome

## Abstract

*Xanthomonas oryzae* pv. *oryzae* (*Xoo*) causes rice bacterial blight (BB), one of the most widespread and destructive diseases in rice-growing regions worldwide. Melatonin enhances pathogen resistance by inducing plant innate immunity, but the direct effect of melatonin on plant pathogenic bacteria is poorly understood. In this study, we investigated the direct effects of melatonin on *Xoo*. Exogenous melatonin at 200 μg/mL significantly inhibited the proliferation of *Xoo* and reduced the mRNA expression of five genes involved in cell division. This concentration of melatonin also inhibited the motility and biofilm formation of *Xoo*. Notably, melatonin was observed to alter the length of *Xoo* cells. To provide deeper insights into the mechanisms underlying this antibacterial activity, we examined global gene expression changes in *Xoo* strain PXO99 in response to the application of 200 μg/mL melatonin using RNA sequencing (RNA-Seq). A wide range of differentially expressed genes (DEGs) related to catalytic activity and metal-binding activity were downregulated in *Xoo* cells in response to the melatonin treatment. In addition, DEGs responsible for carbohydrate and amino acid metabolism were also downregulated. These results suggest that the inhibitory mechanism of melatonin on *Xoo* proliferation may involve the regulation of cell division in combination with a reduction in the concentration or activity of enzymes involved in metabolism.

## Introduction

Melatonin (*N*-acetyl-5-methoxytryptamine) is a highly evolutionarily conserved molecule that exists in the microbe (Manchester et al., 2015), insect (Vivien-Roels et al., 1984), animal (Menendez-Pelaez and Reiter, 1993), and plant kingdoms (Dubbels et al., 1995). In animals, melatonin was discovered in the bovine pineal gland in 1958 (Lerner et al., 1958). This indoleamine is a well-known animal neurohormone involved in numerous cellular and physiological functions, such as sleep (Garfinkel et al., 1995), circadian rhythms (Jung-Hynes et al., 2010), stem cell differentiation (Radio et al., 2006), and scavenging of reactive oxygen species (ROS) and reactive nitrogen species (RNS) (Reiter et al., 2016). In plants, melatonin was simultaneously discovered by two research groups in 1995 (Dubbels et al., 1995; Hattori et al., 1995).

Since then, melatonin has been found in a variety of plant species (Kolar and Machackova, 2005). Plant melatonin is involved in many significant plant processes, including plant growth (Chen et al., 2009; Arnao and Hernández-Ruiz, 2017) and defence against both biotic (Vielma et al., 2014; Shi et al., 2016) and abiotic stresses (Byeon and Back, 2016; Zhang et al., 2017). In microbes, exogenous melatonin acts as a biocide against some fungi and bacteria (Wang et al., 2001; Hu et al., 2017). Melatonin shows antibacterial activity against Gram-positive and Gram-negative bacteria at a low concentration *in vitro* (Atroshi et al., 1998; Konar et al., 2000; Ozturk et al., 2000; Karakas et al., 2013). *In vivo*, the exogenous application of melatonin was shown to suppress *Pst*DC3000 propagation in Arabidopsis leaves (Lee et al., 2014). Melatonin may prevent the uptake of free iron by bacteria (Limson et al., 1998; Tekbas et al., 2008), inhibit constitutive bacterial protein secretion (Bubis and Zisapel, 1995), and reduce intracellular substrates that are important for bacterial growth (Tekbas et al., 2008). However, the mechanisms underlying these inhibitory effects of melatonin on bacteria have been little studied.

Bacterial blight (BB) of rice caused by *X*. *oryzae* pv. *oryzae* (*Xoo*) is one of the most destructive diseases in most rice-growing countries, especially those in Asia (Mansfield et al., 2012). This disease leads to leaf blight during the growing season, hindering photosynthesis and diminishing the production and quality of crops (Mahmood et al., 2006). Despite attempts to control BB by broad-spectrum breeding with high-yield cultivars, this disease remains a major constraint on rice production (Suh et al., 2013). Earlier research demonstrated that *N*-acetylserotonin methyltransferase (ASMT), the last enzyme involved in the synthesis of melatonin, was induced during *Xoo* infection (Wei et al., 2016). However, there have been no reports on the relationship between melatonin and *Xoo*.

In this study, we used *Xoo* strain PXO99 to determine whether melatonin exhibits antibacterial activity against this pathogen. In addition, the relationships between melatonin and cell division and morphology were investigated. We also used RNA sequencing (RNA-Seq) to explore how melatonin, in its role as an antibiotic, inhibits the growth of PXO99. A genome-wide expression profiling analysis clearly demonstrated that many genes involved in metabolic and transcription processes were downregulated. Our results could help to gain a better understanding of the mechanisms by which melatonin inhibits the proliferation of Gram-negative bacteria.

## Materials and Methods

### Bacterial Strain and Plants

*Xoo* strain PX099 was streaked on nutrient agar (NA) medium (beef extract, 3 g/L; yeast extract, 1 g/L; polypeptone, 5 g/L; sucrose, 10 g/L; and agar, 15 g/L) and incubated at 28°C for 2 days. Rice seedlings of the Nipponbare (*Oryza sativa* spp. *Nipponbare*) cultivar were germinated and grown in a growth chamber under an alternating 12-h light, 30°C/12-h dark, 28°C cycle with a photon flux density of 200 μmol/m^2^.s^1^. Rice leaves were inoculated with *Xoo* strain PXO99 (race P6) for pathogenicity tests using the leaf clipping method (Kauffman et al., 1973). Tobacco plants (*Nicotiana benthamiana, Nb*) were grown in a growth chamber under an alternating 12-h light/12-h dark cycle at 25°C with a photon flux density of 120 μmol/m^2^.s^1^. Tobacco leaves were inoculated with PXO99 for hypersensitive reaction (HR) assays using the needleless syringe method (Xu et al., 2015). Statistical analyses were performed using SPSS Version 20.0. The variables were analyzed using Student’s *t*-tests and were tested for significance at the *P* < 0.05 (^∗^), *P* < 0.01 (^∗∗^), *P* < 0.001 (^∗∗∗^), and *P* < 0.0001 (^∗∗∗∗^) levels.

### Measurement of the Effect of Melatonin on Bacterial Growth

*Xoo* strain P6 was incubated with shaking in nutrient broth (NB) medium (NA without agar) at 28°C until an OD_600_ = 1.0 (early logarithmic phase) was reached. The cells were harvested and resuspended in an equal volume of sterilized ddH_2_O. Next, 0.5 mL of the cell suspension was added to 50 mL of NB liquid medium containing different concentrations of melatonin (0, 200, 400, or 1000 μg/mL). Methanol (MeOH) solvent without melatonin (0 μg/mL) served as a control. All cultures were shaken (200 rpm) at 28°C in the dark, and the OD_600_ was measured every 3 h until bacterial growth reached the stationary phase. Each experiment was performed three times, with three replicates per experiment.

### Transmission Electron Microscope (TEM) Observations

The concentration of fresh bacteria in sterilized ddH_2_O was adjusted to OD_600_ = 1.0. Next, 0.5 mL of cell suspension was added into 50-mL fresh NB medium containing different concentrations of melatonin (0, 200, or 400 μg/mL). Methanol (MeOH) solvent without melatonin (0 μg/mL) served as a control. All cultures were grown at 28°C with shaking at 200 rpm for 12 h. Bacterial samples were placed on copper mesh grids with formvar membranes and negatively stained with phosphotungstic acid (2% v/v, pH = 6.7). The samples were then observed using a TEM (Hitachi H-7650) at 80 kV and photographed with a Gatan832 CCD camera (Gatan, Pleasanton, CA, United States).

### Determination of Cell Motility and Biofilm Formation

Swimming motility and biofilm formation assays were performed as described previously (Tian et al., 2015). The concentration of fresh bacteria in sterilized ddH_2_O was adjusted to OD_600_ = 1.0. Next, a 5-μL aliquot of the bacterial suspension was spotted onto semi-solid NA (0.3% agar) containing different concentrations of melatonin (0, 10, 40, or 250 μg/mL). Methanol (MeOH) solvent without melatonin (0 μg/mL) served as a control. Cell motility was monitored after a 96 h incubation at 28°C in darkness. Each experiment was performed three times, with five replicates per experiment. For the biofilm formation assay, a 30-μL cell suspension was inoculated into 3 mL NB liquid medium containing different concentrations of melatonin (0, 10, 40, or 250 μg/mL). After inoculation, the cultures were incubated at 28°C for 5 days without shaking. After gently removing the cultures, the cells adhered to the culture tubes were stained with two volumes of 10% (w/v) crystal violet solution and incubated at 28°C without shaking for 1 h, followed by gentle washing with sterilized ddH_2_O three times, and air drying for 1 h. The crystal violet in the stained cells was dissolved using destaining buffer [40% methanol (v/v), 10% glacial acetic acid (v/v), 50% ddH_2_O (v/v)], and the absorbance at 595 nm (OD_595_) was measured using a spectrophotometer (Eppendorf, Germany). Each experiment was performed three times, with six replicates each time.

### Measurement of Endogenous Melatonin

To determine the melatonin content of *Xoo* cells, a direct sample extraction method was used. The concentration of fresh bacteria in sterilized ddH_2_O was adjusted to OD_600_ = 1.0. Next, 0.5 mL of cell suspension was added to 50 mL of fresh NB medium containing different concentrations of melatonin (0 or 200 μg/mL). Methanol (MeOH) solvent without melatonin (0 μg/mL) served as a control. All cultures were grown at 28°C with shaking at 200 rpm for 24 h. The two cultures were adjusted to the same concentration (OD_600_ = 1.0) and washed with sterilized ddH_2_O three times. Next, the cultures were centrifuged and the pellets were suspended in 10 mL of acetonitrile buffer. The bacterial cells were homogenized using a sonicator (Scientz, Ningbo). After centrifugation, the supernatants were subjected to LC-MS as described previously (Huang and Mazza, 2011).

### RNA Sequencing and Data Analysis

RNA was extracted from strain PXO99 treated with MeOH (M0) and 200 μg/mL melatonin (M200) and used for RNA sequencing. After a 21 h incubation, bacterial cultures at OD_600_ = 1.0 (early logarithmic phase) in broth were harvested. Total RNA was extracted from the mock and melatonin-treated samples using TRIzol reagent (Invitrogen, Carlsbad, CA, United States) according to the procedure recommended by the manufacturer. The following steps were then completed by a commercial company (Genepioneer Biotechnologies Corporation, Nanjing, China). Three micrograms of RNA per sample was used for library construction. For direct comparisons, two libraries (M0 and M200) were prepared in the same manner and sequenced on an Illumina HiSeq Xten platform. We selected genes with a log_2_FC > 2 and *p* < 0.01 for further analysis. Differentially expressed genes (DEGs) between the melatonin-treated and mock samples were analyzed by Gene Ontology (GO) and Kyoto Encyclopaedia of Genes and Genomes (KEGG) enrichment.

### RNA Extraction and Quantitative Real-Time PCR Analysis

Specific primers for quantitative real-time PCR (qRT-PCR) were designed with Primer 5 (version 5) using the corresponding gene sequences from the NCBI database (**Supplementary Table S1**). Total RNA was isolated using TRIzol reagent (Invitrogen, Carlsbad, CA, United States) according to the procedure recommended by the manufacturer, treated with DNase I (Takara, Japan) to eliminate genomic DNA, and then converted into cDNA using a PrimeScript^TM^ RT Reagent Kit (Takara, Japan). Next, qRT-PCR was performed using diluted cDNA and SYBR Green PCR Master Mix (Takara, Japan) on a Quant Studio 6 Real Time PCR system (Thermo Fisher Scientific, United States). The expression data, given as quantification cycle (Cq), were collected and statistically processed using the 2^-Δ(ΔC_p_)^ method. RecA was used as an internal control, and each experiment was conducted three times with three replicates.

## Results and Discussion

### Melatonin Inhibits *Xoo* Growth

**FIGURE 1:**
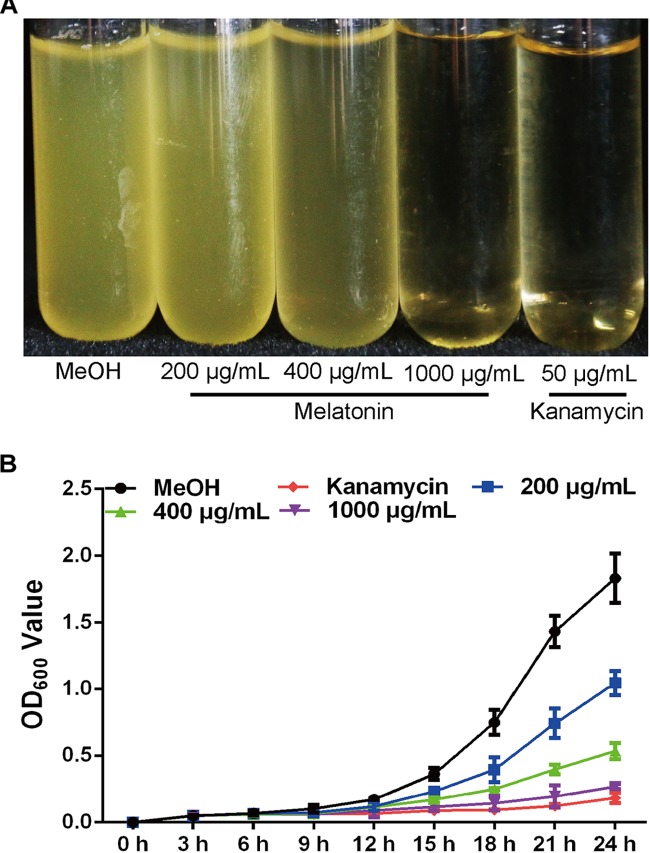
Melatonin inhibits the growth of *Xoo*. **(A)** The state of PXO99 under melatonin treatment. **(B)** Statistical analysis of growth curves of PXO99 treated with different concentrations of melatonin (μg/mL) and cultured for 24 h.

Melatonin has been previously observed to play multiple roles in a wide variety of significant processes in plants, animals, and humans (Dollins et al., 1994; Guerrero and Reiter, 2002; Shi et al., 2015; Fu et al., 2017). However, the impact of melatonin on agriculturally relevant bacteria has not been explored. To this end, we assessed the bacterial growth rates of *Xoo* treated with methanol (mock control) and various concentrations of melatonin (**Figure 1A**). The OD_600_ value of PXO99 at 24 h pre-treated with melatonin (200 μg/mL) was approximately 1.0, only half that of the control group (**Figure 1B**). Thus, 200 μg/mL of melatonin effectively reduced the growth of PXO99. When the concentration was elevated, the bacterial density showed a greater decrease. No PXO99 cells survived 24 h in broth containing 1000 μg/mL melatonin or 50 μg/mL kanamycin. Melatonin represses the growth of human pathogenic bacteria at certain concentrations (Hu et al., 2017), including that of *Streptococcus agalactiae* at 2 μg/mL (Atroshi et al., 1998) and *Saccharomyces cerevisiae* at 1000 μg/mL (Konar et al., 2000). Our growth inhibition results showed that melatonin inhibited PXO99 growth in a concentration-dependent manner, and the inhibitory effect may be dose dependent. In plants, we also observed that 200 μg/mL melatonin suppressed the HR induced by PXO99 on tobacco leaves (**Supplementary Figure S1**). Overexpression of a melatonin-induced gene (*OsMAPK12-1*) resulted in plants with enhanced disease resistance against PXO99 (Xiao et al., 2017). Melatonin-induced plant resistance is mediated by MAPK signaling (Lee and Back, 2017) Thus, whether the suppression of HR caused by melatonin-induced plant innate immunity or melatonin inhibiting the proliferation of PXO99 caused the observed plant disease resistance still requires further investigation.

### Melatonin Reduces *Xoo* Swimming Motility but Increases Biofilm Formation

**FIGURE 2:**
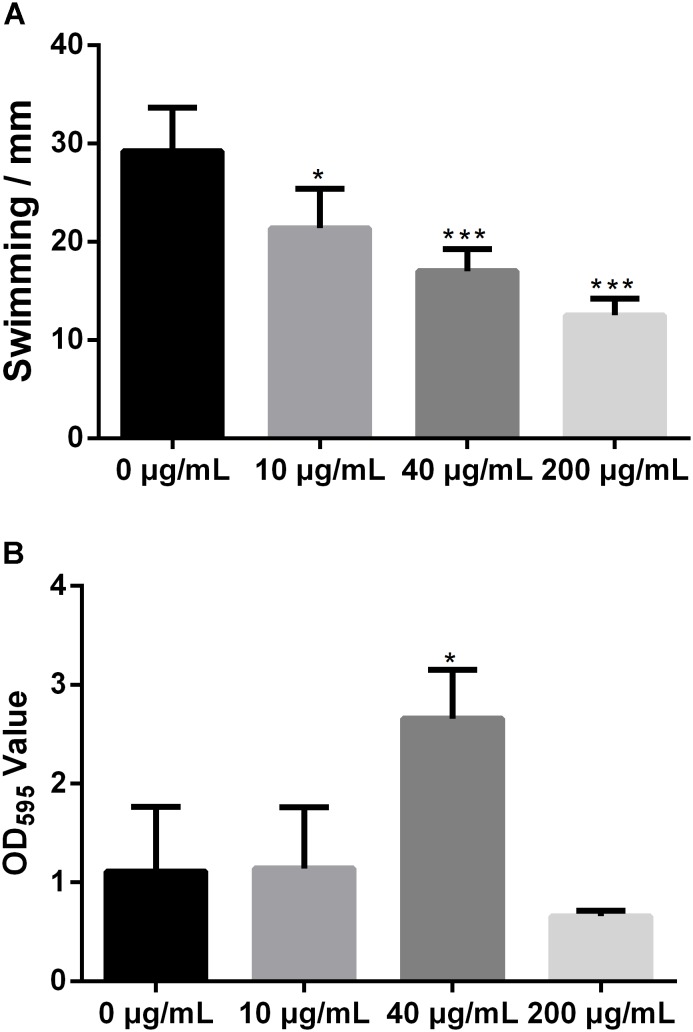
Statistical analysis of the swimming motility **(A)** and biofilm formation of *Xoo* **(B)** treated with melatonin. ^∗^*P* < 0.05, ^∗∗∗^*P* < 0.001.

Swimming motility is necessary for biofilm formation and is crucial for bacterial attachment (O’Toole and Kolter, 1998; de Kerchove and Elimelech, 2008). However, the impact of melatonin on bacterial swimming motility has rarely been reported. To study the influence of melatonin on bacterial motility, the swimming motility diameter of *Xoo* was measured in the presence and absence of melatonin. In initial experiments, we observed that a higher concentration of melatonin disrupted the swimming ability of *Xoo*. Thus, melatonin was used at no more than 200 μg/mL in subsequent tests. As shown in **Figure 2A**, the colony diameters in plates with 10 μg/mL melatonin were decreased by more than 30% compared with that observed in the mock control. When the melatonin concentration was increased, the swimming motility diameter was further decreased. The colony diameters in plates with 200 μg/mL melatonin were reduced by more than half compared with that observed in the mock control. Thus, melatonin affected the motility of *Xoo* in a concentration-dependent manner. These results suggest that the inhibition of bacterial motility by melatonin may occur through increasing cell death, although further investigation of this possibility is necessary.

**Table 1:** Differentially expressed genes in *Xoo* treated with melatonin.

No.	Gene ID	FC^a^	Annotation
**Transcription and translation**			
1	PXO_RS00600	-2.83	Transposase
2	PXO_RS24475	+1.60	Transposase
3	PXO_RS24500	+1.97	Transposase
4	PXO_RS27155	-2.48	Isrso17-ISXo8 transposase protein
5	PXO_RS08305	-1.56	AbrB family transcriptional regulator
6	PXO_RS05275	-2.34	LysR transcriptional regulator
7	PXO_RS17440	-1.84	TetR family transcriptional regulator
8	PXO_RS09765	-2.27	PhoU family transcriptional regulator
9	PXO_RS08695	-1.98	GntR family transcriptional regulator
10	PXO_RS19870	-2.38	PhoB family transcriptional regulator
11	PXO_RS20365	-1.91	AraC family transcriptional regulator
12	PXO_RS13760	-1.88	MarR family transcriptional regulator
13	PXO_RS23615	-3.61	Alkaline phosphatase D
14	PXO_RS23610	-4.78	Alkaline phosphatase
15	PXO_RS18050	-2.20	DNA-binding protein
16	PXO_RS08310	-1.56	DNA-binding protein
17	PXO_RS03000	-1.60	Disulphide-isomerase
18	PXO_RS20265	-2.92	Chlamydia polymorphic membrane family protein
19	PXO_RS07715	-1.80	MFS transporter
**Carbon and protein metabolism**			
20	PXO_RS22365	-2.39	Acetyl-CoA acetyltransferase
21	PXO_RS12655	-2.11	Acetyl-CoA acetyltransferase
22	PXO_RS11725	-2.01	Acetyl-CoA acetyltransferase
23	PXO_RS22370	-2.39	3-Oxoadipate:succinyl-CoA transferase, partial
24	PXO_RS01865	2.03	3-Methylcrotonyl-CoA carboxylase subunit alpha
25	PXO_RS11720	-2.07	3-Hydroxyacyl-CoA dehydrogenase
26	PXO_RS12650	-2.07	3-Hydroxyacyl-CoA dehydrogenase
27	PXO_RS20635	-1.90	2-Methylisocitrate lyase
28	PXO_RS20105	-2.20	Malate dehydrogenase
29	PXO_RS02070	-2.19	NADH-dependent FMN reductase
30	PXO_RS27075	+1.86	Fumarylacetoacetate hydrolase domain protein
31	PXO_RS08315	-2.97	Bifunctional aconitate hydratase 2/2-methylisocitrate dehydratase
32	PXO_RS21360	-2.23	Chorismate mutase
33	PXO_RS20590	-4.82	Glycerophosphodiester phosphodiesterase
34	PXO_RS00340	-2.69	Aldolase
35	PXO_RS00350	-2.69	Aldolase
36	PXO_RS23355	-1.69	Cellulase
37	PXO_RS05615	-3.88	Xylanase
38	PXO_RS01665	-2.32	Xylose isomerase
39	PXO_RS01605	-4.72	Beta-1,4-xylanase
40	PXO_RS19450	+2.54	Glycosidases
41	PXO_RS19890	-2.48	Glycosyl transferase
42	PXO_RS21065	-1.75	Mannose-1-phosphate guanyltransferase
43	PXO_RS23055	+1.61	Fucose permease
44	PXO_RS19900	-2.48	UDP-2,3-diacylglucosamine hydrolase
45	PXO_RS18550	-1.50	Ubiquinol oxidase subunit II
46	PXO_RS22130	-2.61	Lipase
47	PXO_RS15625	-2.94	Peptidase S53
48	PXO_RS06470	-2.30	Peptidase C1
49	MSTRG.1600	+1.94	Pentapeptide repeats family protein
50	PXO_RS16310	-3.64	Oar protein
**Signal transduction**			
51	PXO_RS19895	-2.48	Phosphoesterase
52	PXO_RS05010	-1.93	Phosphoanhydride phosphohydrolase
53	PXO_RS09785	-2.66	Phosphate-binding protein
54	PXO_RS09790	-2.35	Phosphate-binding protein
55	PXO_RS09775	-1.88	Phosphate transporter permease subunit PtsA
56	PXO_RS09780	-1.88	Phosphate ABC transporter permease
57	PXO_RS09770	-2.44	Phosphate ABC transporter ATP-binding protein
58	MSTRG.1547	-2.42	Putative ABC transporter phosphate-binding protein
59	PXO_RS17230	-2.63	Sulfite reductase
60	PXO_RS17465	-1.51	Serine/threonine protein kinase
61	MSTRG.60	-1.83	Serine kinase
62	MSTRG.2902	-1.51	Cytochrome D ubiquinol oxidase subunit II, partial
63	PXO_RS18555	-1.50	Cytochrome bd-type quinol oxidase, subunit 1
**Cell structure**			
64	PXO_RS13030	+1.50	Flagellar biosynthesis
65	PXO_RS12100	+1.68	Flagellar biosynthesis
66	PXO_RS00345	-2.69	Flagellar biosynthesis protein FliP
67	PXO_RS12815	+1.63	Flagellar basal body rod protein FlgB
68	PXO_RS11885	+1.76	Flagellar basal body rod protein FlgB
**Pathogenicity**			
69	PXO_RS19875	-2.08	Two-component system sensor protein
70	PXO_RS08560	-2.18	Type VI secretion protein
71	PXO_RS08540	-2.18	Type VI secretion protein
72	PXO_RS00365	-2.69	Type III secretion system protein
73	PXO_RS23030	-2.97	Type III secretion system effector protein
74	PXO_RS02310	-2.84	Type III secretion system effector protein
75	PXO_RS03830	-2.24	Type III secretion system effector protein
76	PXO_RS00355	-2.69	Type III secretion protein
77	PXO_RS08565	-2.18	Tal3b, TAL effector AvrBs3/PthA family
78	PXO_RS08545	-2.12	Tal3a, TAL effector AvrBs3/PthA family
79	PXO_RS00740	-4.91	Tat pathway signal protein
80	MSTRG.3198	-1.97	TonB-dependent receptor
81	PXO_RS19075	-4.14	TonB-dependent receptor
82	PXO_RS20595	-3.71	TonB-dependent receptor
83	PXO_RS20360	-1.91	TonB-dependent receptor
84	MSTRG.2688	-2.54	TonB-dependent receptor, partial
85	PXO_RS00735	-3.43	TonB-dependent receptor
86	PXO_RS17235	-2.63	TonB-dependent receptor
87	PXO_RS00360	-2.69	Hypersensitivity response secretion protein hrcV
88	PXO_RS00320	-3.97	HrpE
89	PXO_RS00370	-3.09	HPr kinase
90	PXO_RS00330	-2.69	HPr kinase
91	PXO_RS00325	-2.66	HPr kinase
92	PXO_RS00375	-2.63	HPr kinase
93	PXO_RS00315	-3.13	protein HpaB
94	PXO_RS00335	-2.69	Protein HpaA
95	PXO_RS06005	-2.01	Putative sulfotransferase required for AvrXa21 activity ST (raxST)
96	PXO_RS25330	-1.75	Xanthomonadin biosynthesis protein
97	PXO_RS21615	-2.04	Adhesin
98	PXO_RS22720	+1.51	Ankyrin
99	PXO_RS22730	+1.58	Hemolysin D
**Stress response**			
100	PXO_RS05270	-2.49	Protocatechuate degradation protein
101	PXO_RS05265	-2.24	Protocatechuate 3,4-dioxygenase subunit beta
102	PXO_RS22360	-1.98	Protocatechuate 3,4-dioxygenase subunit beta
103	PXO_RS22355	-1.75	Protocatechuate 3,4-dioxygenase subunit alpha
104	PXO_RS13750	-2.23	Multidrug transporter
105	PXO_RS08690	-1.65	Multidrug transporter
106	PXO_RS13755	-1.88	Multidrug RND transporter
107	emrB	-1.68	Multidrug resistance protein B
108	PXO_RS27000	-2.18	Multidrug resistance efflux pump
109	PXO_RS09795	-2.78	Porin
**Function unknown**			
110	PXO_RS25045	-3.49	Hypothetical protein
111	PXO_RS24050	-2.67	Hypothetical protein
112	PXO_RS26160	+1.56	Hypothetical protein
113	PXO_RS25955	+1.52	Hypothetical protein
114	PXO_RS00415	-4.29	Hypothetical protein
115	PXO_RS19070	-4.12	Hypothetical protein
116	PXO_RS00605	-3.49	Hypothetical protein
117	PXO_RS00425	-3.28	Hypothetical protein
118	PXO_RS00580	-3.09	Hypothetical protein
119	PXO_RS22960	-3.07	Hypothetical protein
120	PXO_RS20695	-3.03	Hypothetical protein
121	PXO_RS01615	-2.32	Hypothetical protein
122	PXO_RS20585	-2.18	Hypothetical protein
123	PXO_RS00380	-2.10	Hypothetical protein
124	PXO_RS01795	-1.95	Hypothetical protein
125	PXO_RS03845	-1.84	Hypothetical protein
126	PXO_RS09760	-1.73	Hypothetical protein
127	PXO_RS03505	-1.64	Hypothetical protein
128	PXO_RS02075	-1.50	Hypothetical protein
129	PXO_RS22735	+1.62	Hypothetical protein
130	PXO_RS17460	-2.95	Hypothetical protein
131	PXO_RS17455	-2.95	Hypothetical protein
132	PXO_RS06000	-2.85	Hypothetical protein
133	PXO_RS21520	-2.36	Hypothetical protein
134	PXO_RS17445	-2.33	Hypothetical protein
135	PXO_RS06495	-2.32	Hypothetical protein
136	PXO_RS01735	-1.60	Hypothetical protein
137	PXO_RS25040	-2.83	Hypothetical protein
138	PXO_RS25015	-2.62	Hypothetical protein


*^*a*^The symbol “+” indicates upregulated genes in *Xoo* cells treated with melatonin, while the symbol “-” indicates downregulated genes in *Xoo* cells treated with melatonin.*

Biofilm formation plays a crucial role in plant pathogen infections (Parsek and Singh, 2003). Melatonin was reported to inhibit the biofilm formation of *Candida parapsilosis* and *S. aureus* ATCC29213 at 2.9 and 340 μg/mL, respectively (Yang et al., 2014; Romic et al., 2016). Biofilm-associated pathogens can form light-colored rings on the wall of a culture tube at the interface between air and broth. To evaluate the effect of melatonin on the attachment of *Xoo*, the biofilm formation of PXO99 in response to melatonin challenge was analyzed. As shown in **Figure 2B**, the presence of 10 μg/mL melatonin slightly increased the biofilm formation of PXO99. When the melatonin concentration was increased, the CV absorbance at OD_595_ showed a greater increase. The observed OD_595_ value from tubes containing 40 μg/mL melatonin was threefold higher than that of the mock control. However, the opposite effect was observed when melatonin was present at higher concentrations. The OD_595_ value in the tubes containing melatonin at 200 μg/mL was 40% lower than that of the control. Thus, the effects of melatonin on PXO99 biofilm formation did not resemble the observed effects on swimming motility or growth inhibition. When melatonin was present at a high concentration, no swimming motility, or biofilm formation was observed. Our results suggest that melatonin may induce biofilm formation in *Xoo* at low concentrations but inhibit its formation at high concentrations. Interestingly, we observed that both the bacterial abundance and lesion length in rice leaves infected with PXO99 treated with melatonin (200 μg/mL) was similar to that of the control group (**Supplementary Figure S2**). Moreover, the HR in tobacco leaves induced by PXO99 treated with melatonin (200 μg/mL) was similar to that of the control group (**Supplementary Figure S3**). Thus, the results indicated that melatonin may not affect *Xoo* pathogenicity.

### *Xoo* Becomes Highly Enriched With Melatonin

**FIGURE 3:**
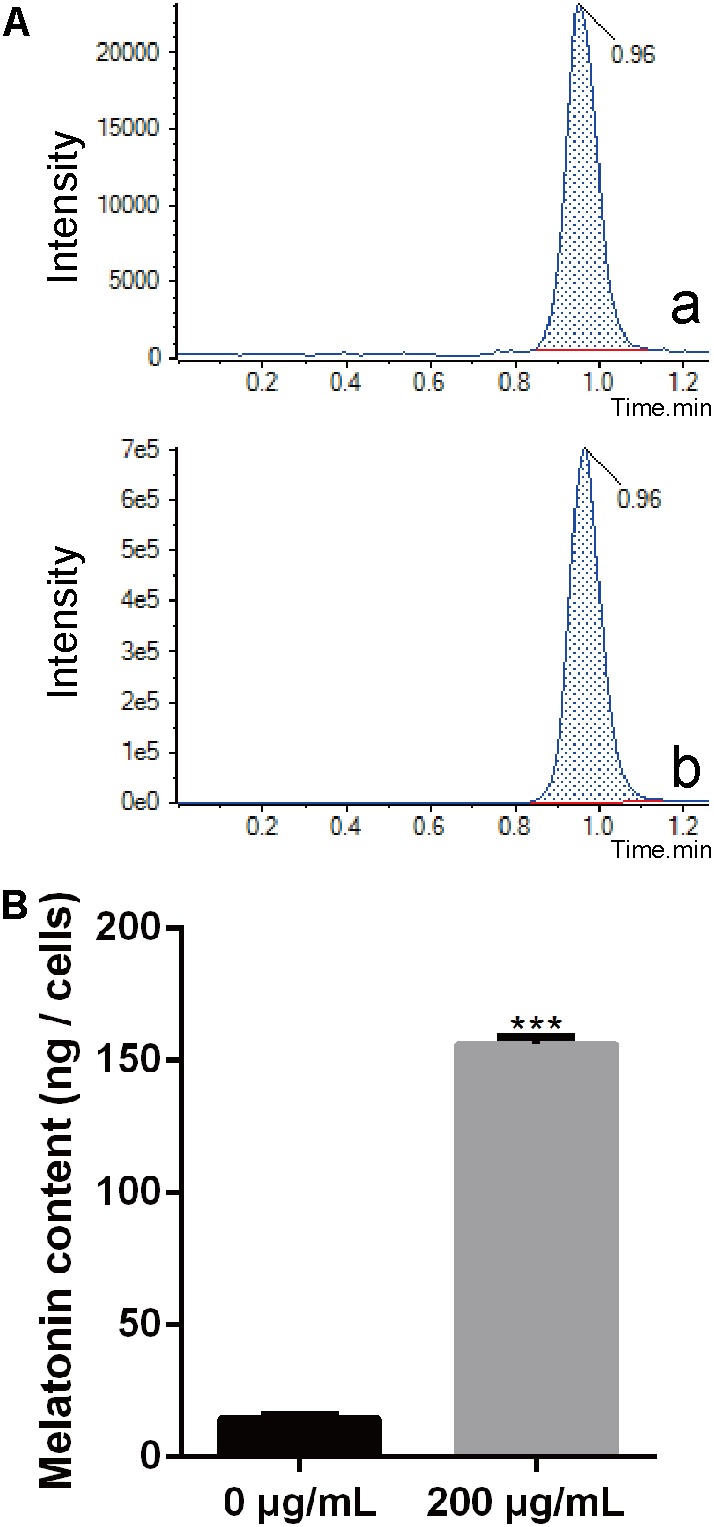
Extraction and identification of melatonin in *Xoo*. **(A)** Chromatogram corresponding to melatonin. **(a)** Chromatogram corresponding to melatonin collected from PXO99 cells not treated with melatonin (Sigma). **(b)** Chromatogram corresponding to melatonin collected from PXO99 cells pre-treated with melatonin (Sigma). **(B)** Statistical analysis of melatonin (ng/cells) from PXO99 cells. UV response: 280 nm. ^∗∗∗^*P* < 0.001.

Melatonin has been observed to easily pass through cell walls (Tekbas et al., 2008). In this study, we evaluated the content of endogenous melatonin in PXO99 cell treated with melatonin by LC-MS. The endogenous melatonin was 14.43 ng in POX99 cells that were harvested from 30 mL broth cultures after incubating for 24 h. In contrast, 156.13 ng of endogenous melatonin was detected in POX99 cells that were incubated with exogenous melatonin and harvested from 30 mL broth cultures after incubating for 24 h (**Figure 3A**). The results showed that melatonin can easily pass through the cell wall and become enriched in *Xoo* cells. The endogenous melatonin detected in the treatment group was approximately 100 times that in the control group (**Figure 3B**). This disruption in normal endogenous melatonin levels in *Xoo* may inhibit the proliferation of this bacterium. Because melatonin was detected in PXO99, we can assume that *Xoo* has the ability to synthesize melatonin and may have a biosynthetic pathway that is similar to that present in plants or animals. However, the function of melatonin in *Xoo* needs further study.

### Melatonin Inhibits *Xoo* Cell Division

**FIGURE 4:**
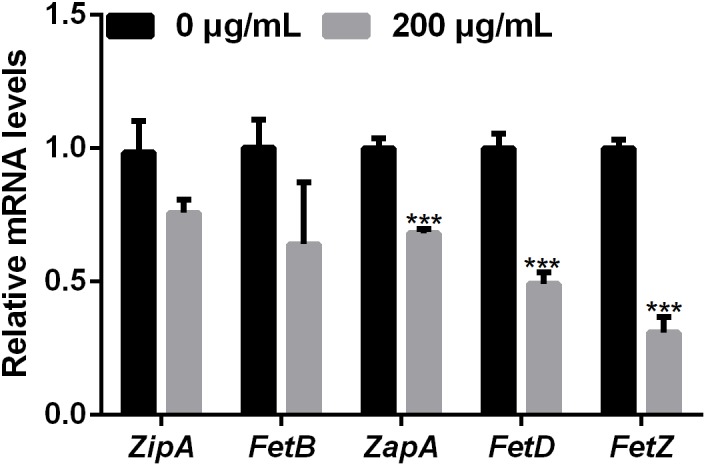
qRT-PCR analysis of the mRNA expression of cell-division-related genes in *Xoo* treated with melatonin. ^∗∗∗^*P* < 0.001.

Bacterial cell division occurs by the formation of a Z-ring at the site of division (Lutkenhaus and Addinall, 1997). The dynamics of the Z-ring are regulated by the cell division-related genes *ZapE* and *FtsZ*, and the role of ZapA is to recruit *ZapB* to the inner face of the Z-ring (Galli and Gerdes, 2010; Marteyn et al., 2014). To investigate whether melatonin inhibits bacterial proliferation by disrupting or inhibiting cell division, the mRNA expression of nine cell division-related genes in PXO99 challenged with melatonin (200 g/mL) was analyzed by qRT-PCR. The mRNA expression of many internal genes has been reported to be affected by melatonin treatment (Sheshadri et al., 2018). In preliminary experiments, we tested the stability of two PXO99 internal candidate reference genes and observed that *RecA* was the more stable of the two in PXO99 cells treated with melatonin. As shown in **Figure 4**, four cell division-related genes (*FetQ, ZapE, FetL*, and *FetE*) were upregulated, and five (*ZipA, FetB, ZapA, FetD*, and *FetZ*) were downregulated in *Xoo* cells treated with melatonin compared to the control cells. Our results indicate that the melatonin treatment resulted in a decrease in *Xoo* cell division. Because bacterial proliferation depends on the ability of cells to divide (Pardee, 1989), the reduction in cell division could result in an inhibition of *Xoo* growth.

### Melatonin Alters *Xoo* Morphology

**FIGURE 5:**
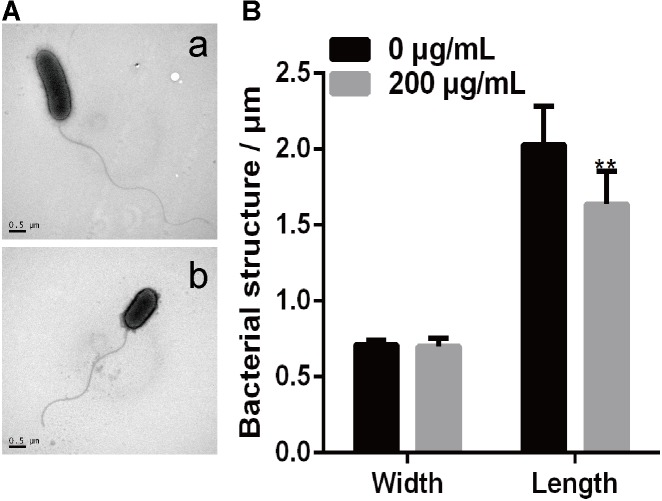
Observations of *Xoo* morphology following treatment with melatonin. **(A)** The morphology of PXO99 cells. **(a)** The morphology of PXO99 cells not treated with melatonin. **(b)** The morphology of PXO99 cells treated with melatonin (200 μg/mL). **(B)** Statistical analysis of the width and length of PXO99 cells. Bar = 0.5 μm.

A previous study showed that *P*. *infestans* cells treated with melatonin exhibited reduced lipid droplet production and inhibited the proliferation of *P*. *infestans* (Zhang et al., 2017). In this study, we investigated the effect of melatonin on the cellular morphology of PXO99 by making TEM observations. As shown in **Figure 5**, both bacterial size and shape were easily distinguished by TEM using a negative staining method. The width and length of individual PXO99 cells ranges from 0.6 to 1.0 μm and from 1.0 to 2.7 μm, respectively, and our observations agreed with these specifications (**Figure 5A**). By contrast, the width of PXO99 cells treated with melatonin was slightly shorter than in the control, and the length of PXO99 treated with 200 μg/mL of melatonin exhibited a significant reduction (20%) compared to the control (**Figure 5B**). These data indicate that the reduction in the cell length of PXO99 treated with melatonin may result from the inhibition of *Xoo* proliferation.

### RNA-Seq Transcriptome Analysis of Melatonin-Treated *Xoo*

**FIGURE 6:**
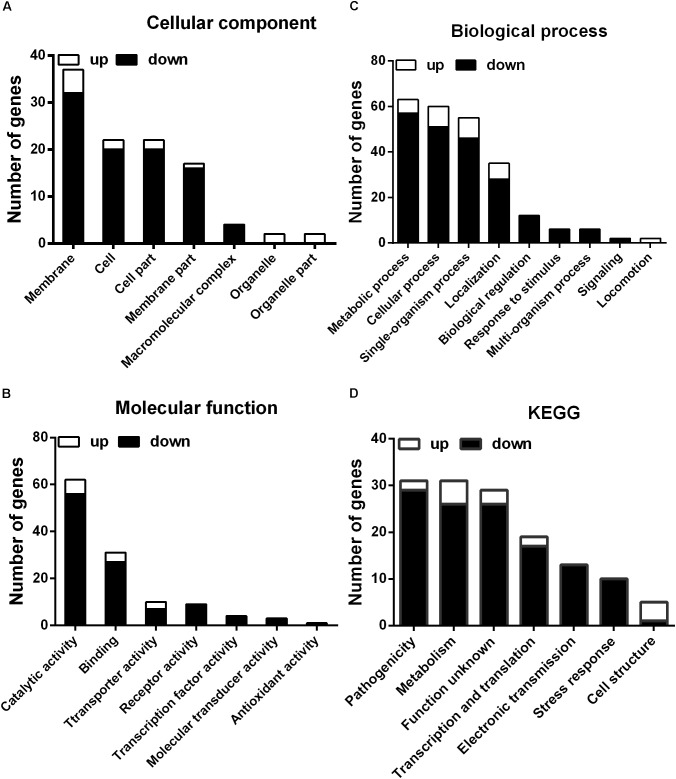
Classification of differentially expressed genes by gene ontology (GO) enrichment and cellular mapping. **(A)** Number of differentially expressed genes. **(B)** Cellular component classifications. **(C)** Molecular function. **(D)** Biological process.

**FIGURE 7:**
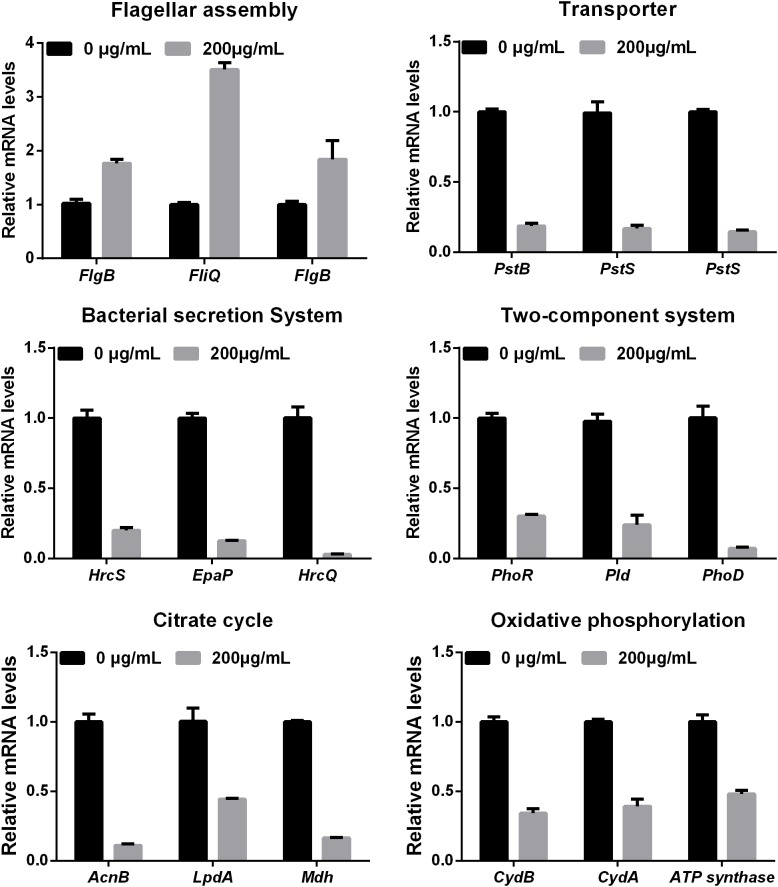
Validation of 18 differentially expressed genes at the mRNA level by qRT-PCR.

To further investigate the mechanism of the effects of melatonin on *Xoo* proliferation, total RNA from PXO99 cells that were treated or untreated with melatonin was collected and analyzed by RNA-Seq. An analysis of the gene expression changes obtained from the RNA-Seq assay showed that 138 genes had alterations in mRNA transcript levels in response to a melatonin challenge at 21 h post-treatment (**Table 1**), corresponding to 2.73% of the *Xoo* genome. Fourteen genes were upregulated, and 124 genes were downregulated, and these DEGs were characterized both by using the GO database, which provides annotation information regarding cellular components, molecular functions and biological processes, and by using the KEGG database. Of the 14 upregulated genes, four were enriched in flagellar components, four were enriched in transporter activity, and three were involved in metabolic processes Flagella are used for motility in PXO99. Whether the four flagellar genes regulated by melatonin are involved in swimming motility or biofilm formation requires further study. Transporters are well known to play a crucial role in substantial exchanges between cells and the outside environment, and the upregulated genes involved in transporter activity and metabolic processes may help PXO99 survive. Among the observed downregulated genes, a notable overrepresentation of genes associated with membrane and cellular components was observed in the cellular component category (**Figure 6A**). Moreover, genes encoding catalytic activity-related proteins were overrepresented in the molecular function category (**Figure 6B**). Consistently, a notable overrepresentation of genes associated with metabolic processes in the biological processes category was observed (**Figure 6C**). In the metabolic processes, 41 genes were dominant in the biological processes category (**Figure 6C**). Consistently, 56 and 27 genes involved in catalytic activity and metal-binding activity, respectively, were dominant in the category of molecular functions (**Figure 6B**). To verify the reliability of the transcriptomes, 18 randomly selected genes were analyzed using qRT-PCR. The results were consistent with the sequencing data (**Figure 7**). Genes related to oxidative phosphorylation, citrate cycle, protein secretion, and two component systems were downregulated in PXO99 treated with melatonin.

### Melatonin Regulates *Xoo* Metabolism

**FIGURE 8:**
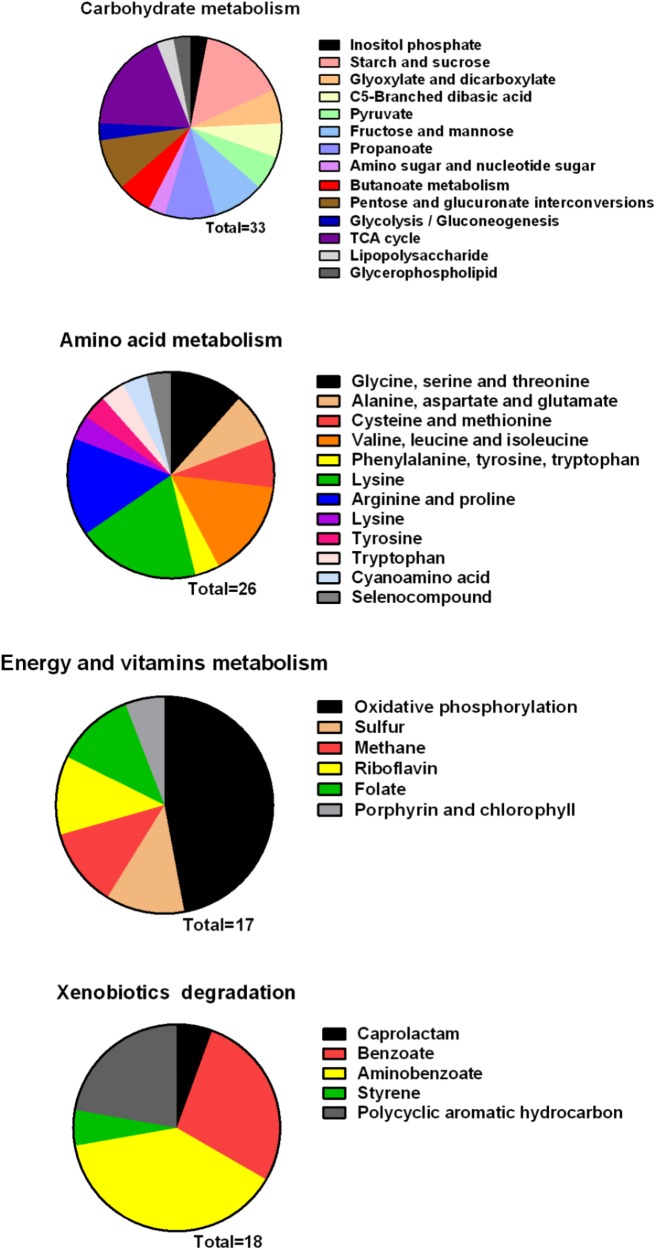
Classification of differential genes in the metabolism in PXO99 under melatonin treatment.

Metabolism is an important characteristic of bacteria, and melatonin was reported to significantly reduce the expression mRNA of genes associated with metabolism in microbes (Zhang et al., 2017). In this study, we observed that genes involved in carbohydrate and amino acid metabolism were enriched (**Figure 8**). The best carbon and nitrogen sources for *Xoo* growth are sucrose and glutamate (Singh, 2016). Interestingly, we observed that many genes involved in sucrose and glutamate metabolism were downregulated in PXO99 when challenged with melatonin.

Bacterial pathogens are known to require iron for replication and infection (Schaible and Kaufmann, 2004; White and Yang, 2009; Skaar, 2010). *Xoo* requires ferrous sulfate for optimal proliferation and modulates copper redistribution in rice during infection (Yuan et al., 2010). According to the RNA-Seq results, the mRNA expression of the transporter TonB, which is responsible for iron absorption (Yue et al., 2003), was downregulated in PXO99 challenged with melatonin (**Table 1**). Other DEGs related to metal binding were also downregulated (**Figure 6B**). The mRNA levels of genes that encode metal-ion binding and cation binding proteins were previously observed to be downregulated in rice leaves treated with melatonin, similar to our results (Liang et al., 2015). The content of endogenous melatonin in melatonin-treated PXO99 was approximately 100 times that of the control group (**Figure 3**). Interestingly, 18 genes involved in xenobiotic metabolism were downregulated (**Figure 8**). Melatonin has a strong ability to bind copper and iron(III) (Limson et al., 1998). Thus, we speculate that melatonin can cause a free iron deficiency in bacterial cells and inhibit growth through the metal-binding activity of melatonin or by reducing the concentration and activity of metal-binding enzymes.

Phosphate is most commonly used in energy metabolic processes and serves as a buffering agent in cells (Lardy and Wellman, 1952). In this study, the mRNA expression of the transcription factor PhoU, which function in environmental phosphate (Pi) sensing and transportation (Muda et al., 1992), was reduced in PXO99 cells treated with melatonin (**Table 1**). In addition, DEGs encoding proteins located on the cell membrane related to phosphate transporter and phosphate binding proteins involved in energy metabolism were both downregulated (**Figure 8**). In humans, melatonin inhibits cancer cell growth by preventing the cell membrane from assimilating linoleic fatty acid (Blask et al., 1999). The results of this study indicated that the inhibitory mechanism of melatonin on bacterial growth may be related to reducing phosphate levels, although a detailed characterization of these mechanisms will require further investigation.

## Conclusion

In this study, we investigated the potential effects of melatonin on *X*. *oryzae* pv. *oryzae*. Our data showed that melatonin can cross the cell wall and become enriched in *Xoo* cells, inhibiting the cell division and proliferation of this bacterium. Importantly, melatonin altered the cell structure and reduced the motility and attachment ability of *Xoo* cells. The results of the transcriptome analysis suggest that the inhibitory effects of melatonin on *Xoo* proliferation may occur through (i) decreasing cell division and (ii) reducing the concentration and activity of enzymes involved in metabolism. This work provides new insights into the inhibitory effect of melatonin on bacterial growth and gene expression.

## Author Contributions

FL, ZF, and XC designed the study. XC and CS performed the experiments. XC, CS, and YZ analyzed the data. XC, CS, and PL drafted the manuscript. FL, IP, JQ, and ZF reviewed and edited the manuscript.

## Conflict of Interest Statement

The authors declare that the research was conducted in the absence of any commercial or financial relationships that could be construed as a potential conflict of interest.

## Footnotes

1 http://www.genome.jp/kegg/

## Supplementary Material

FIGURE S1 The hypersensitive reaction triggered by *Xoo* on tobacco leaves pretreated with melatonin. The tobacco leaves were inoculated with different concentrations of *Xoo* (ddH_2_O, OD_600_ = 0.2, 0.4, 0.8). **(a)** Tobacco leaves without the melatonin treatment and **(b)** tobacco leaves pretreated with 200 μg/mL melatonin for 12 h.

FIGURE S2 Effects of exogenous melatonin on the pathogenicity of PXO99 and the hypersensitive response. **(A)** Phenotype of rice leaves inoculated with PXO99 treated with melatonin (200 μg/mL). **(B)** Lesion length on rice leaves inoculated with PXO99 treated with melatonin (200 μg/mL). **(C)** Bacterial population in rice leaves inoculated with PXO99 treated with melatonin (200 μg/mL). Bar = 2 cm.

FIGURE S3 The hypersensitive reaction of tobacco leaves triggered by *Xoo* pretreated with melatonin. *Xoo* cells were pretreated with melatonin (200 μg/mL) for 24 h. **(A)** The tobacco leaves inoculated with ddH_2_O, *Xoo* without melatonin treatment, *Xoo* pretreated with melatonin (200 μg/mL). **(B)** The mRNA expression of HrpD6 in response to the melatonin treatment.
