# Supplementary figures and images for: Melatonin Treatment Inhibits the Growth of Xanthomonas oryzae pv. oryzae
Source: Front Microbiol. 2018 Oct 4;9:2280. doi: 10.3389/fmicb.2018.02280 (PMC6180160; doi:10.3389/fmicb.2018.02280)

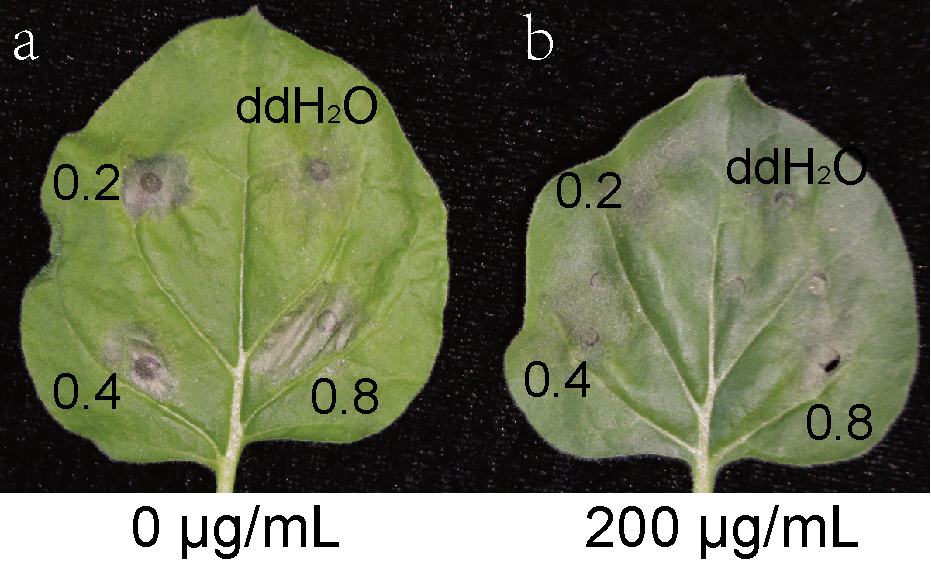

Supplement: FIGURE S1 — The hypersensitive reaction triggered by Xoo on tobacco leaves pretreated with melatonin. The tobacco leaves were inoculated with different concentrations of Xoo (ddH2O, OD600 = 0.2, 0.4, 0.8). (a) Tobacco leaves without the melatonin treatment and (b) tobacco leaves pretreated with 200 μg/mL melatonin for 12 h. [file Image_1.TIF]

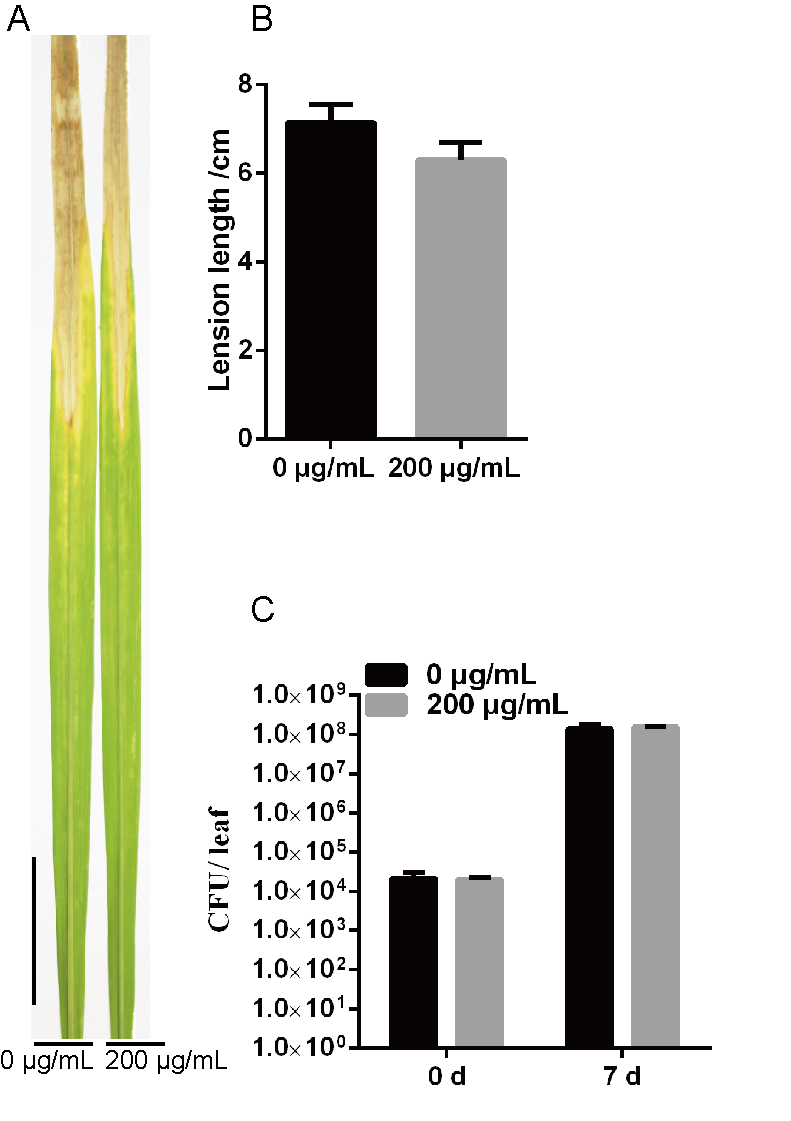

Supplement: FIGURE S2 — Effects of exogenous melatonin on the pathogenicity of PXO99 and the hypersensitive response. (A) Phenotype of rice leaves inoculated with PXO99 treated with melatonin (200 μg/mL). (B) Lesion length on rice leaves inoculated with PXO99 treated with melatonin (200 μg/mL). (C) Bacterial population in rice leaves inoculated with PXO99 treated with melatonin (200 μg/mL). Bar = 2 cm. [file Image_2.TIF]

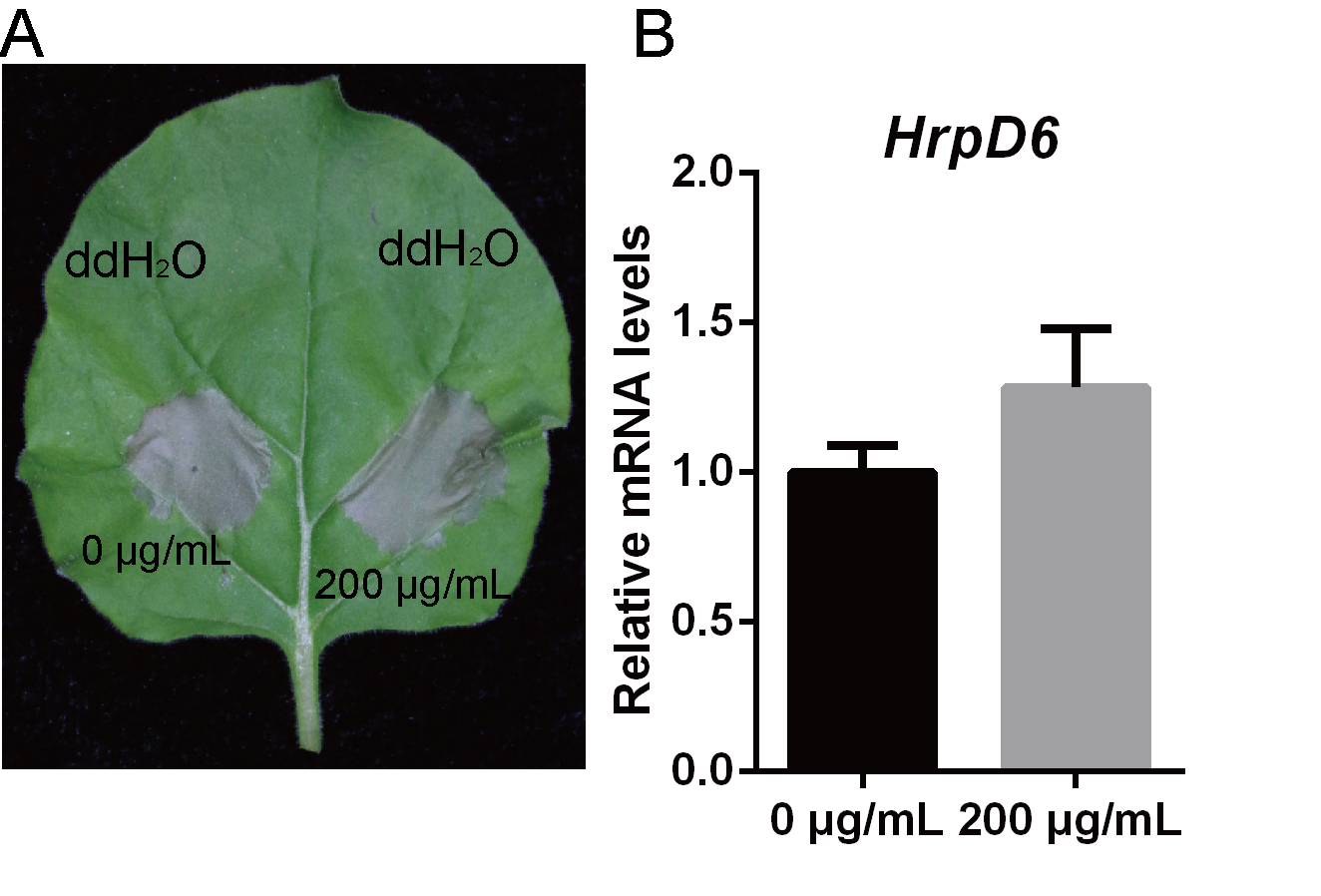

Supplement: FIGURE S3 — The hypersensitive reaction of tobacco leaves triggered by Xoo pretreated with melatonin. Xoo cells were pretreated with melatonin (200 μg/mL) for 24 h. (A) The tobacco leaves inoculated with ddH2O, Xoo without melatonin treatment, Xoo pretreated with melatonin (200 μg/mL). (B) The mRNA expression of HrpD6 in response to the melatonin treatment. [file Image_3.TIF]
