# Supplementary material for: Melatonin Treatment Inhibits the Growth of Xanthomonas oryzae pv. oryzae
Source: Front Microbiol. 2018 Oct 4;9:2280. doi: 10.3389/fmicb.2018.02280 (PMC6180160; doi:10.3389/fmicb.2018.02280)
Supplement: Supplementary file 4 [file Data_Sheet_1.doc]

**TABLE S1│Primers used for RT-PCR to detect the mRNA of DGEs**

| Gene ID | Protein name | RT-PCR Primer ( 5’ to 3’) | Products (bp) |
| --- | --- | --- | --- |
| *XO_RS09770* | *PstB* | F:CGCATCTTCAACCGCATCTACGC | 144 |
|  |  | R:AACGGCACCGGCTTCTGGAACACC |  |
| *PXO_RS09785* | *PstS* | F:CCGCCCTGTCCCTTACCAT | 188 |
|  |  | R:CGGAGATTTCAGCACCCTTG |  |
| *PXO_RS09790* | *PstS* | F:CTGGAAGAGCAAGGTGGGC | 164 |
|  |  | R:ACGACAGCTCGACGTAGCC |  |
| *PXO_RS00340* | *HrcS* | F:CGACGATCTAGTGCGATTTACC | 243 |
|  |  | R:GACATCACTGCCTGGACGAA |  |
| *PXO_RS00345* | *EpaP* | F:CAATTCCTCCTCAAGCATACAC | 125 |
|  |  | R:ACACCAGATACAACAGAAACCC |  |
| *PXO_RS00350* | *HrcQ* | F:ACGCCAATGCGGCTTCGT | 153 |
|  |  | R:CGTCGTCGTCCTCGTTCAATAA |  |
| *PXO_RS19875* | *PhoR* | F:CTGACGGTGGTGCATGGCTATCTG | 172 |
|  |  | R:TGCTCTTCGCCGAGTTCTTCCTG |  |
| *PXO_RS23610* | *Pld* | F:CAGGGCTTCGGCATCAATC | 167 |
|  |  | R:CAGCGTTTCGGCCACCTT |  |
| *PXO_RS23615* | *PhoD* | F:GACGACCACGAGGTACAGAACG | 121 |
|  |  | R:TCCAGCGAGCGGTAGATGC |  |
| *PXO_RS08315* | *AcnB* | F:TGGTGTTCGATGCGTTCCA | 218 |
|  |  | R:CAGGTCGTCGGTGTTGGTTT |  |
| *PXO_RS13570* | *lpdA* | F:CGCTCAAGGGCACAAACG | 152 |
|  |  | R:GAACGGCGGTGAAATCCAG |  |
| *PXO_RS20105* | *Malate dehydrogenase* | F:TGCGTCTGGACCATAACCG | 242 |
|  |  | R:GGTCGGAATGAAGGTGGATG |  |
| *PXO_RS18550* | *CydB* | F:GGCGTGGTTTGCGGTGAT | 208 |
|  |  | R:AGGTTTCGTTGCCGTCCC |  |
| *PXO_RS18555* | *CydA* | F:CCGTTGCTGAGCTATGAAGTG | 125 |
|  |  | R:GAGAATCCAGAACGTGGAGACC |  |
| *PXO_RS21395* | *ATP synthase* | F:TGGCAAGTTCCTGGAATCGG | 204 |
|  |  | R:TTGGCGAAGGCGAACAGC |  |
| *PXO_RS06005* | *RaxST* | F:CCAGCCGTTGCGTTTGTC | 144 |
|  |  | R:CAGCATGGTCGCCGTAGAA |  |
| *PXO_RS06010* | *Hemolysin D* | F:CGAACTGCGTCGTCGTTTCC | 151 |
|  |  | R:CGTCCATCTCCAGCCTTAGCG |  |
| *PXO_RS06015* | *ABC transporter* | F:CGAACTGCGTCGTCGTTTCC | 160 |
|  |  | R:CGTCCATCTCCAGCCTTAGCG |  |
| *PXO_RS11885* | *FlgB* | F:CCGGCTACAAGGCCAAAGA | 184 |
|  |  | R:CCGTCAAGGCTGGGCTGAT |  |
| *PXO_RS12100* | *FliQ* | F:CCTGAAATCGCCCTGACTG | 150 |
|  |  | R:GCAATGGTCGGCTCGTTC |  |
| *PXO_RS12815* | *FlgB* | F:CCGGCTACAAGGCCAAAGA | 184 |
|  |  | R:CCGTCAAGGCTGGGCTGAT |  |
| *PXO_RS00325* | *HrpD6* | F: CGATGCGGTCACTCAGGATAT | 163 |
|  |  | R: CTGGCGATATGCACGACGAT |  |
| *PXO_RS07900* | *RecA* | F:AATGCCTTGAAGTTCTACGCC | 141 |
|  |  | R:TTCGGTCACGACCTGCTTG |  |


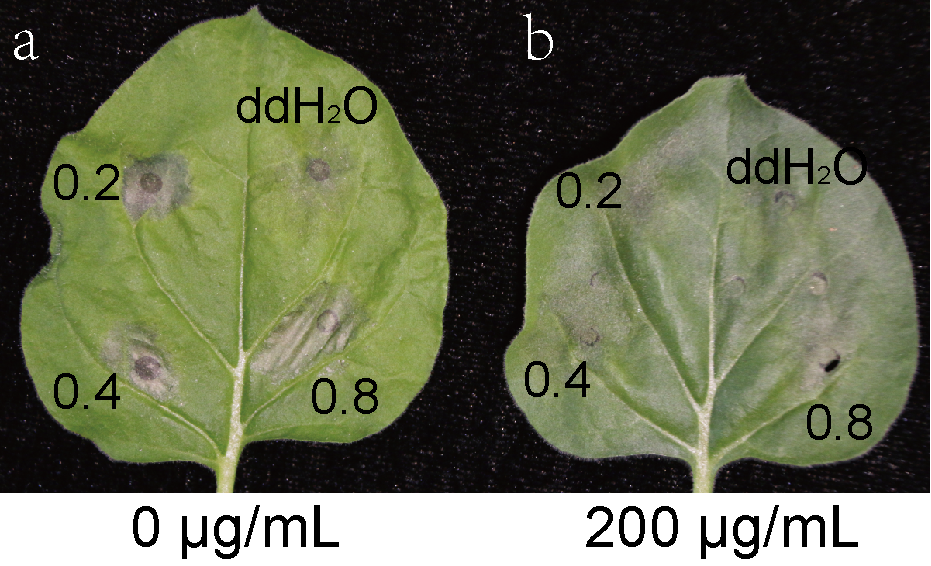


**FIGURE S1**│ The hypersensitive reaction triggered by *Xoo* on tobacco leaves pretreated with melatonin. The tobacco leave was inoculation with different concentration of *Xoo* (ddH_2_O, OD_600_=0.2, 0.4, 0.8). (**a**: tobacco leaves without melatonin treatment, **b**: tobacco leaves pretreated with 200 μg/mL melatonin for 12 h)
